# Minimal acupuncture is not a valid placebo control in randomised controlled trials of acupuncture: a physiologist's perspective

**DOI:** 10.1186/1749-8546-4-1

**Published:** 2009-01-30

**Authors:** Iréne Lund, Jan Näslund, Thomas Lundeberg

**Affiliations:** 1 Department of Physiology and Pharmacology, Karolinska Institutet, Stockholm, Sweden; 2 Foundation for Acupuncture and Alternative Biological Treatment Methods, Sabbatsbergs Hospital, Stockholm, Sweden

## Abstract

Placebo-control of acupuncture is used to evaluate and distinguish between the specific effects and the non-specific ones. During 'true' acupuncture treatment in general, the needles are inserted into acupoints and stimulated until *deqi *is evoked. In contrast, during placebo acupuncture, the needles are inserted into non-acupoints and/or superficially (so-called minimal acupuncture). A sham acupuncture needle with a blunt tip may be used in placebo acupuncture. Both minimal acupuncture and the placebo acupuncture with the sham acupuncture needle touching the skin would evoke activity in cutaneous afferent nerves. This afferent nerve activity has pronounced effects on the functional connectivity in the brain resulting in a 'limbic touch response'. Clinical studies showed that both acupuncture and minimal acupuncture procedures induced significant alleviation of migraine and that both procedures were equally effective. In other conditions such as low back pain and knee osteoarthritis, acupuncture was found to be more potent than minimal acupuncture and conventional non-acupuncture treatment. It is probable that the responses to 'true' acupuncture and minimal acupuncture are dependent on the aetiology of the pain. Furthermore, patients and healthy individuals may have different responses. In this paper, we argue that minimal acupuncture is not valid as an inert placebo-control despite its conceptual brilliance.

## Background

Randomised placebo-controlled clinical trials (placebo-controlled RCTs) are used to evaluate the efficacy of medical interventions. The ultimate intention of these placebo-controlled RCTs is to eliminate the non-specific placebo effects [1]. This trial design is considered as the gold standard. The results of placebo-controlled RCTs provide evidence for a treatment's efficacy [2]. However, the technical issues in developing valid placebos in acupuncture RCTs are still controversial [1,3-7].

### Placebo

The placebo concept was introduced into RCTs as a treatment without curative anticipation [8]. Randomised, double-blind, placebo-controlled trials are generally considered as the best experimental method for separating the 'specific' from the 'non-specific placebo related' effects of a treatment. The placebo is supposed to be inert, inducing only non-specific physiological and emotional changes. If the intervention is a drug, the 'specific' component is the pharmacologically active agent while the placebo is an inert substance. Recent studies have, however, shown that some placebos are sometimes therapeutically effective [9]. The issue of evaluation becomes more complicated especially if the intervention in question is as complex as acupuncture [7,10]. Acupuncture may be viewed from a Chinese medicine perspective whereby each acupoint is associated with specific effects, or from a Western perspective whereby acupuncture is merely what its Latin name suggests – 'acus' (needle) and 'pungere' (to prick), and its effects are explained in Western physiological terms.

#### Localisation: Chinese medicine versus physiological aspects

In Chinese medicine, the correct acupoints are vital in the classical theory of acupuncture to achieve efficacy. A possible control intervention from this perspective is, therefore, needling at incorrect sites. From a physiological perspective, an acupoint is defined by its anatomical innervation. Needling at an incorrect site may affect the correct receptive field in terms of physiology. In such a scenario, the physiological responses to needling at incorrect sites may be identical.

#### Needling effects: Chinese medicine versus physiological aspects

In Chinese medicine, depths of needling, manipulation of the needle, triggering of a specific irradiating needling sensation known as *deqi *(considered to be associated with effective needling), duration of stimulation may all vary according to a holistic diagnosis. From a physiological perspective, acupuncture is a modality of sensory stimulation and the effects obtained are dependent on which sensory receptors are activated, the afferent activity set-up and the resulting activity in the central nervous system. The response of the nervous system to the sensory input is dependent on its present state and also on the characteristics of the individual (e.g. genotype, coping strategy, expectation and previous experiences). Given the complexity, it is not surprising that a variety of control interventions have been used in clinical acupuncture trials. Dincer and Linde reviewed the sham-controlled clinical trials of acupuncture, particularly on (a) which sham interventions were used, (b) in what respects 'true' and sham interventions differed and (c) whether trials using different types of sham yielded different results [10]. They included 47 randomised controlled trials published in English or German in which trial patients received either 'true' acupuncture or sham (referred to as 'sham' or 'placebo') for preventive, palliative or curative purposes. The sham interventions used were categorized as follows.

I: superficial needling of 'true' points (superficial needling of the acupoints for the treated condition)

II: 'irrelevant' acupoints (needling of the acupoints not for the treated condition)

III: 'non-acupuncture' points (needling non-acupoints)

IV: 'placebo needles' (devices that mimic acupuncture without skin penetration)

V: pseudo-interventions (interventions that are not 'true' acupuncture e.g. use of switched-off laser acupuncture devices)

Dincer and Linde also examined whether the 'true' and sham interventions differed in terms of points chosen, penetration of the skin, depths of needling, manipulation or stimulation of the needle, achievement of *deqi*, number of points, number of sessions and duration of sessions. Out of the 47 included trials, two trials employed the sham intervention that consisted of superficial needling of the 'true' acupuncture points; four trials used 'true' acupoints not indicated for the condition being treated; in 27 trials needles were inserted outside 'true' acupoints; five trials used placebo needles and nine trials used pseudo-interventions such as switched-off laser acupuncture devices. 'True' and sham interventions often differed in other aspects, such as manipulation of needles, depth of insertion, and achievement of *deqi *and there was no clear association between the type of sham intervention used and the results of the trials. Dincer and Linde concluded that considering all these different sham interventions as simple 'placebo' controls was misleading and scientifically unacceptable [10].

### Effects of minimal acupuncture

A technique defined as minimal acupuncture may be used as a control to acupuncture. The number, length, and frequency of the sessions in the minimal acupuncture are the same as for the 'true' acupuncture. Typically, at least five out of 10 predefined distant non-acupuncture bilateral points (at least 10 needles) are needled superficially in each session. Furthermore, manual stimulation of the needles and *deqi *is avoided. Even if this may be a valid control from the Chinese medicine perspective, it is not necessarily from a physiological perspective.

#### Stimulus intensity

In chronic pain patients with sensitisation of the peripheral and central nervous systems, the acupuncture stimulus response is augmented, whereby light stimulation of the skin, minimal acupuncture may have an effect as strong as acupuncture in various integrated physiological responses [11]. Central sensitisation is also associated with expanded receptive fields of central neurons, resulting in a larger topographic distribution of the pain [12]. This suggests that control procedures with light needling of the skin and/or needling away from the target treatment site (area of pain), in patients with central sensitisation, may have effects equivalent to needling within the treatment site [13]. In patients who do not suffer from central sensitisation, repeated nociceptive input from muscles (as obtained in *deqi*) results in expansion of receptive fields which may in turn lead to activation of descending pain inhibition outside the stimulated myotome [11]. In other words, a control procedure with needling in a nearby myotome may have similar effects as needling within the affected myotome. An increased sensitivity to pain, and other sensory modalities, may be related to abnormalities in descending efferent pathways and plasticity changes in the nervous system, thereby influencing the effects of acupuncture [14-16].

#### Aetiology and characteristics of pain

Depending on the characteristics of the pain, e.g. spontaneous, persistent or stimulus-evoked and its related default mode, acupuncture may have different effects [11,13,17,18]. Furthermore, the aetiology of the clinical condition or syndrome must be considered for appropriate design of the control procedure [19-23]. Otherwise, optimal pain inhibition may not be achieved [19].

### Physiological complexity of acupuncture effects

#### Pain inhibition

There are various kinds of modern and traditional approaches to acupuncture treatment [23,24]. Depending on the approach, different results may be obtained [25,26]. It has been postulated that acupuncture analgesia, in the case of manual acupuncture, is manifested by the feeling of *deqi*. During manual acupuncture, all types of afferent nerve fibres (A-beta, A-delta and C) can be activated while minimal acupuncture (with needles applied superficially into the skin) probably activates two types of C tactile fibres in the skin [27-32]. Electro-acupuncture results in activation of A-beta- and part of A-delta nerve fibres in response to the stimulating current delivered to acupuncture points via the inserted needle. The nerve impulses, emanating from the acupuncture stimulation, ascend mainly through the spinal ventrolateral funiculus to the brain. Many brain nuclei of an integrated network are involved, including the periaqueductal grey, nucleus raphe magnus, arcuate nucleus, preoptic area, locus coeruleus, accumbens nucleus, nucleus submedius, caudate nucleus, habenular nucleus, septal area and amygdale [33-37]. These areas are also involved in emotional and reward processes.

It was shown that various endogenous systems played crucial roles in acupuncture analgesia, for example, the systems that involve activation of endogenous opioids (beta-endorphin, enkephalin, endomorphin and dynorphin) and the desending serotoninergic inhibitory pathway [35]. The functions of these systems altered according to the aetiology of the pain. Apart from endogenous opioids and serotonin, the cholecystokinin octapeptide (CCK-8) was shown to play a key role in the effects of acupuncture including development of tolerance [37]. The individual differences of acupuncture analgesia are also associated with inherited genetic factors and the density of CCK receptors. Furthermore, acupuncture analgesia is probably associated with its counter-regulation of spinal glial activation, PTX-sensitive Gi/o protein-mediated and MAP kinase-mediated signal pathways, and downstream processes [36].

#### Self- appraisal

The brain modulates processes involved in self-appraisal during acupuncture. For example, when a patient sees an acupuncturist, there is anticipation of a specific effect [38-43]. This anticipation is partly based on self-relevant phenomena and self-referential introspection that will constitute the preference. These self-appraisal processes are dependent on two integrated networks, namely a ventral medial prefrontal cortex-paralimbic-limbic 'affective' pathway and a dorsal medial prefrontal cortex-cortical-hippocampal 'cognitive' pathway [44].

#### Limbic structures and reward

The limbic structures show an increased activity in most diseases and illness responses [45-48]. Acupuncture including electro-acupuncture and minimal acupuncture may result in deactivation of limbic structures (in patients with pain) [49-53]. Deactivation of limbic structures has been associated with an increased activity in hypothalamus and the resulting activation of pain and sympathetic inhibiting mechanisms [54]. Not only does the brain modulate the activity in the hypothalamus and the limbic structures, but also modulates the reward system resulting in a sensation of wellbeing during acupuncture [44]. Acupuncture may work as behavioural conditioning, which suggests that the needling procedure *per se *may have therapeutic effects [55].

### Minimal acupuncture in migraine, low back pain and knee osteoarthritis pain

**Figure 1:**
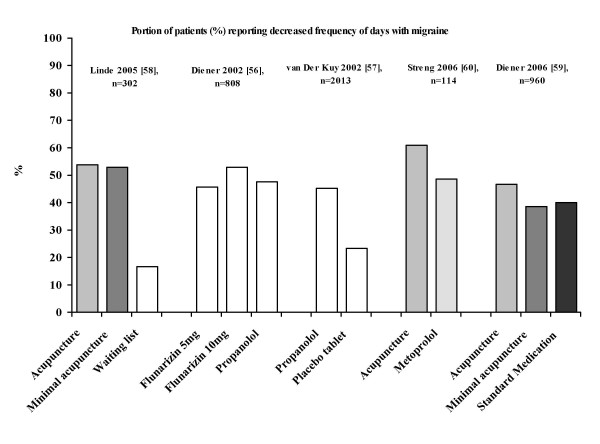
**Reported respondent rates across recent trials of migraine treated with various interventions**. Respondents were defined as those who reported reduction of pain. The figure was modified from a PowerPoint presentation [6] with the permission of Dr M Cummings.

**Figure 2:**
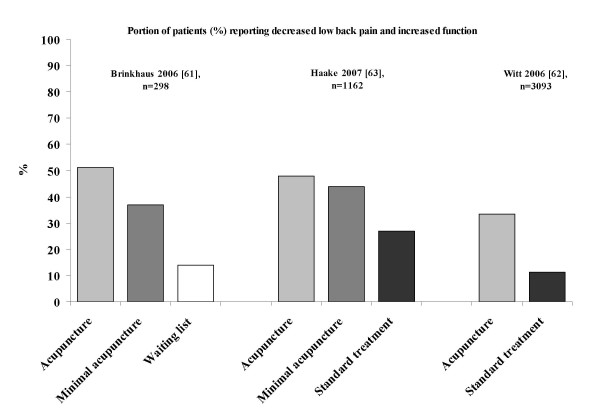
**Reported respondent rates across recent trials of low back pain treated with various interventions**. Respondents were defined as those who reported increased function. The figure was modified from a PowerPoint presentation [6] with the permission of Dr M Cummings.

**Figure 3:**
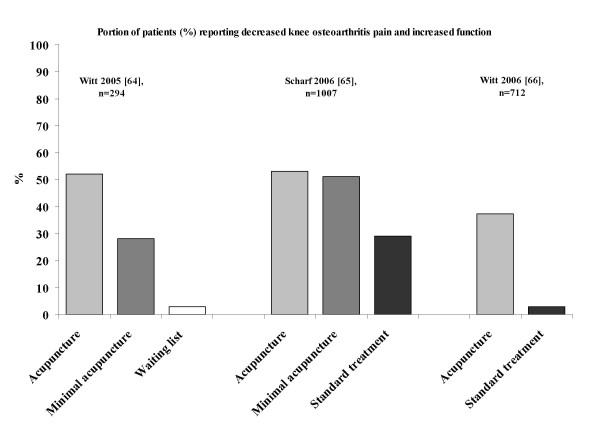
**Reported respondent rates across recent trials of knee osteoarthritis pain treated with various interventions**. Respondents were defined as those who reported increased function. The figure was modified from a PowerPoint presentation [6] with the permission of Dr M Cummings.

It was suggested that both acupuncture and minimal acupuncture may induce activation of sensory afferents [7,11,27-32]. The relevant question is whether minimal acupuncture of the skin has a clinical effect. If it does, the present research paradigm (acupuncture versus placebo with minimal acupuncture) is not valid. This suggestion is illustrated in Figures 1, 2, 3 based on the studies of the efficacy of acupuncture in migraine (Figure 1), low back pain (Figure 2) and knee osteoarthritis pain (Figure 3) [56-66]. The results of the above studies showed that minimal acupuncture had therapeutic effects. Clinically, both 'true' acupuncture and minimal acupuncture are effective in migraine, whereas 'true' acupuncture is more effective than minimal acupuncture in low back pain and knee osteoarthritis pain [67].

From the studies of the efficacy of acupuncture in migraine, low back pain and knee osteoarthritis pain [55-66], an intriguing finding was the strong and lasting response to minimal acupuncture and the lack of significant differences between 'true' acupuncture and minimal acupuncture. This indicates that point location and other aspects considered relevant in Chinese medicine do not make a major difference. However, the improvement over, and the differences compared with, the waiting list group are clearly clinically relevant. The minimal acupuncture intervention used was, according to the investigators, designed to minimise potential physiological effects by needling superficially at points distant from acupoints as well as by using fewer needles (but still at least 10) than 'true' acupuncture. From a physiological perspective, the effects of superficial needling at the points distant from acupoints may still induce a wide range of peripheral, segmental and central physiological responses and in this respect the minimal acupuncture technique is not inert and can therefore not serve as a control for those using acupuncture in a physiological perspective (as a modality of sensory stimulation). An explanation for the improvements observed is that the effects of acupuncture and minimal acupuncture are associated with particularly potent placebo effects. Some evidence shows that complex medical interventions or medical devices have higher placebo effects than placebo drugs [4,5]. Acupuncture treatment has characteristics that are considered relevant in the context of placebo effects. It has an 'exotic' conceptual framework with an emphasis on the 'individual as a whole'. It is associated with frequent patient-practitioner contacts, and it includes the repeated 'ritual' of needling. Finally, the high expectations of patients and the way the patients were informed were demonstrated to be relevant factors in the German trials [67]. From a physiological perspective, however, these so called placebo responses of the acupuncture procedure may be obtained after conditioning and Pavlovian extinction.

### Specific and non-specific effects of minimal acupuncture in clinical conditions – a plausible scenario

A part of the specific effects of minimal acupuncture may be attributed to the deactivation of limbic structures and modulation of default mode [17,68-78]. If it is the case, needle depth or site of stimulation is not essential for eliciting some of the specific effects of acupuncture [79-84]. However, in knee osteoarthritis, minimal acupuncture did not result in the same improvement as acupuncture for the first three months. It is possible that reducing the activity in the limbic structures may restore functional connectivity, making the patient receptive to his or her expectancy of a treatment's effect (specific) and to the patient-therapist interaction (non-specific effect), i.e. the specific effects of minimal acupuncture conditions the non-specific ones [85-90]. Repeated treatment can result in Pavlovian deconditioning/extinction of, for example, knee osteoarthritis pain [91,92]. In such a scenario, the construction of a placebo control is virtually impossible, as any kind of sensory stimulus may have a specific effect. Many acupuncture RCTs did not consider these aspects and therefore led to false negative results. Systematic reviews (e.g. Cochrane studies) and meta-analyses based on the RCTs with false negative results may wrongly conclude that acupuncture has no specific therapeutic effects.

### Other aspects of acupuncture treatment

It is important to emphasise that acupuncture is not a simple needling intervention. There are at least three other processes, apart from needling, that characterize the acupuncture procedure, namely (1) building a treatment relationship, (2) individualizing care and (3) facilitating active engagement of patients in their own recovery [93-95]. These processes include establishing rapport, facilitating communication throughout the period of care, using an interactive diagnostic process, matching treatment to the individual patient and using explanatory models to aid the development of a shared understanding of the patient's condition and to motivate lifestyle changes that reinforce the potential for a recovery of health [96,97]. In a sense, acupuncture requires cognitive behavioural research to further characterize its treatment process.

### Minimal acupuncture as a complement and the use of an observational study protocol

In a recent study [98], researchers investigated the effectiveness of acupuncture combined with the routine medical care in patients with primary headache compared with the treatment of routine care only. Furthermore, they evaluated whether the effects of acupuncture varied in randomised and non-randomised patients. In a three-month follow-up, the number of days with headache was decreased in both acupuncture and control groups. Similarly, the decrease of pain intensity and quality of life improvements were more pronounced in the acupuncture group than that in the control group. Treatment success was maintained throughout the six-month follow-up. The outcome changes in non-randomised patients were similar to those in randomised patients. Patients in acupuncture plus routine care showed marked clinical improvements compared to those with routine care only. These results showed that acupuncture may be demonstrated as a (cost-effective) complement to routine care without using minimal acupuncture as a control. On the other hand, the use of observational study with the data carefully collected over time as events occur, as in a longitudinal study, instead of conventional RCTs, may allow a trial design that suits the clinical situation better [99,100] and avoid inherent difficulties in patient information regarding the sham [101].

## Conclusion

Randomised, placebo-controlled clinical trials of acupuncture are recommended for the evaluation of its efficacy with the goal of separating the specific effects from the non-specific ones. However, it is difficult to define acupuncture control [102]. Experimental and clinical studies have shown that minimal acupuncture, used as placebo control, is not necessarily inert from a physiological perspective. The relevance of using minimal acupuncture as placebo acupuncture must therefore be questioned [103,104]. Instead of reducing bias, this trial design may introduce a bias against the treatment being tested [5]. Therefore, the results obtained from this method should be interpreted with care, particularly under the conditions that minimal acupuncture may have both specific and non-specific effects [105].

## Competing interests

The authors declare that they have no competing interests.

## Authors' contributions

TL drafted the manuscript for discussion. JN and IL contributed their views and revised the manuscript. IL integrated all views and finalised the manuscript. All authors read and approved the final version of the manuscript.
